# Relationship and mechanisms between internet use and physical exercise among middle- and younger-aged groups

**DOI:** 10.1371/journal.pone.0305131

**Published:** 2024-07-03

**Authors:** Hao Chen, Tingpimei Zhang, Yihao Li, Weifeng Zhao, Wei Xu

**Affiliations:** 1 General Graduate School, Dongshin University, Naju, JeollaNamdo, South Korea; 2 Food and Health Research Center, Wuhan Polytechnic University, Wuhan, Hubei, China; 3 School of Economics, Wuhan Polytechnic University, Wuhan, Hubei, China; 4 Center for Counyt Economic Development Research in Hubei, Wuhan Polytechnic University, Wuhan, Hubei, China; University of Study of Bari Aldo Moro, ITALY

## Abstract

The “Internet Plus” era has established a closer connection between sports and individuals. This study used data from the 2018 China Family Panel Studies and focused on the middle- and younger-aged population aged 15 to 59 years. Employing a negative binomial regression model, this study empirically analyzed the impact of Internet use on physical exercise and its internal mechanisms among this population. The findings revealed that (1) Internet use significantly promoted physical exercise in the middle- and younger-aged population, with the frequency of physical exercise increasing to 1.549 times the original value; (2) The positive effects of the internet on physical exercise outweighed the negative effects, with online learning and entertainment enhancing physical exercise and online socialization limiting it. Specifically, online learning and entertainment increased the frequency of physical exercise among the middle- and younger-aged population by 0.063 and 0.018, respectively. Online socialization reduced the frequency by 0.023; and (3) The influence of internet use on physical exercise varies; significantly, it positively affects the exercise frequency among individuals over 35 years old and shows a positive correlation with employment status, including both employed individuals and those out of the labor market. The positive role of Internet use in encouraging physical exercise participation among the middle- and young-aged groups should be valued and enhanced.

## 1. Introduction

One of the most controversial topics in sports is engaging more diverse populations in physical exercise and identifying effective mechanisms of engagement. Data from the “National Fitness Plan (2021–2025)” released by the State Council of China show that in 2020, 37.2% of the Chinese population participated in physical exercise regularly [1]. In countries with well-developed public sports, this rate has not yet reached 50% [2, 3]. Globally, public sports development still faces challenges, such as lack of scientific guidance and inadequate facilitie [4]. The fact remains that effectively improving the population’s health is a key challenge faced by all countries [5].

In this digital age, the Internet has revolutionized not only production methods but also aspects such as information acquisition, entertainment, and leisure. Consequently, the considerable debate regarding the relationship between Internet use and physical exercise has been ongoing in academia. Some scholars have argued that the Internet promotes individual physical exercise engagement. Firstly, the internet has broadened the channels through which individuals can gain knowledge about physical exercise [6]. During the coronavirus disease 2019 (COVID-19) pandemic period, more than half of the population obtained fitness information and participated in physical exercise through the Internet [7, 8]. Secondly, individuals can utilize online fitness instructions, the metaverse, and other means to transcend time and space limitations to exercise [9–11]. However, some scholars have held an opposite view, arguing that considering time duration, the displacement effect of the COVID-19 pandemic has led to an increasing number of the population spending considerable time on the internet [12]. Regarding efficiency, Chinese residents generally lack the skills and knowledge to effectively navigate the digital domain [13]. According to the 52nd “China Internet Development Status Report,” the number of Chinese netizens reached 1.079 billion as of June 2023 [14]. Prolonged watching of short videos increases individual sedentary behavior, which reduces exercise and sleep time [15].

Based on the “2020 Survey on the Status of National Fitness Activities” released by China, people who perform at least 30 minutes of moderate- to high-intensity exercise three or more times weekly are considered people who perform physical exercise regularly [16]. Among middle-aged and senior citizens (aged 50–70 years) in China, 31% engage in physical exercise regularly, a rate that reduces to 14% among the younger- and middle-aged groups [14]. These age groups constitute approximately 70% of China’s total population and are the main users of the Internet as well as those who mostly lack physical activity. The relationship and contradiction between Internet use and physical exercise are especially evident in this demographic. Therefore, this study attempted to explore the following two questions based on the dual nature of the Internet: Does Internet use positively affect physical exercise among the middle- and younger-aged populations? And if an inherent relationship exists between internet use and physical exercise in this demographic, what are the underlying mechanisms?

Therefore, this study used data from the 2018 China Family Panel Studies (CFPS), focusing on the middle- and younger-aged population aged 15 to 59 years and employing a negative binomial regression model to analyze the impact of internet use on physical exercise. The study also explored and validated the mechanisms through which the Internet influences physical exercise.

The remainder of this paper is arranged as follows. Section 2 reviews the related literature. Section 3 describes the data and methods used. Section 4 presents empirical results. Section 5 discusses the findings, and Section 6 outlines the limitations. Section 7 offers the conclusions.

## 2. Literature review and theoretical framework

The literature review on the impact of Internet use on residents’ physical exercise was conducted based on two perspectives. 1. Considering the heterogeneous analysis of subsequent data, the first part of this review mainly focuses on the impact of residents’ individual circumstances, such as their income, education level, and socioeconomic status, on physical exercise. 2. Considering the impact of the Internet on exercise capital and residents’ psychology, the second part of this review discusses factors such as the Internet’s marginal cost, long tail effect, and attachment effect.

The current prevailing view in academia is that the factors influencing physical exercise are divided into three categories including individual, sporting program, and environmental factor. Individual factors refer to the characteristics of an individual that directly affect their exercise behavior. The "endowment-self-causation" dichotomy in social stratification theory divides the individual factors affecting physical exercise into two main categories: "Ascribed factors" and "Achieved factors" [17]. “Ascribed factors” are innate characteristics that cannot be modified, such as gender, family, etc. “Achieved factors” include traits that can be modified, such as education, income, and profession [18]. Regarding gender, the physiological disadvantages and distinct social and familial roles of women compared with men result in their lower participation in physical exercise [19]. The level of participation in physical exercise declines with age due to the dopaminergic system’s regulatory mechanism that affects the motivation for sports participation [20]. Regarding family, the higher the social class of the family, the more parents invest financially and energetically in their children’s physical exercise, leading to a better cultivation of their children’s physical exercise interest and habits [21]. Regarding economic income, the higher an individual’s income, the greater the likelihood of that individual participating in physical exercise. Regarding occupation, white-collar workers compared with blue-collar workers and the general populace have a higher rate of participation in sports [22, 23]. Pertaining educational level, education attainment fosters the development of healthy exercise habits and grants access to more social resources [24].

Sports apps can promote participation in physical exercise and the formation of exercise habits [25]. Emerging virtual reality exercises can help increase exercise frequency and enhance performance among other benefits [26]. While research describes the positive impact of the Internet on physical exercise, some studies have shown that Internet use is not conducive to personal participation in sports, or they are unrelated. Considering the early Internet days, computer use reduced young people’s free time and was negatively correlated or not correlated with physical activity [27, 28]. As research progressed, children and adolescents with higher Internet usage frequencies were identified to have reduced participation in physical exercise [29], and their sedentary behavior increased by more than 50% [30, 31]. High-frequency Internet use does not only limit the time for physical exercise but also affects one’s physical and mental health negatively [32].

The following mechanisms underlie the influence of Internet use on physical exercise.

Firstly, the Internet promotes physical exercise by encouraging individuals to learn about physical exercise. Utilizing Internet learning platforms, people can overcome the temporal and spatial limitations of physical exercise and engage in sports activities during fragmented times in the day [33]. The Internet reduces the marginal time cost of learning physical exercise methods, fulfilling individuals’ diverse needs for physical exercise. Jeremy Rifkin’s marginal cost theory posits that based on a nearly zero marginal cost of information, the total cost of information services is amortized [34]. The advent of the Internet has significantly reduced the marginal cost of producing and distributing sports information products and can almost achieve zero marginal cost [35, 36]. Therefore, the sports industry makes sports knowledge, which could not be effectively promoted in real life due to lack of funds and resources, readily accessible through online sharing platforms [37, 38].

Secondly, the widespread dissemination of information through the Internet has unleashed the diverse leisure and fitness attributes of sports. The “Internet + Sports” model has optimized the public path for sports participation and promoted offline physical exercise behaviors [39]. Chris Anderson’s Long Tail theory posits that a sufficient combination of many niche products can create a market that rivals the mass market [40]. In Internet dissemination, the broadcasting of mass and niche sports coexist [41]. As consumer demands diversify, the status of niche group broadcasting increases [42]. The Internet has resolved the issue of information asymmetry between sports consumers and producers, enabling the precise targeted promotion of diversified and refined sports resources and achieving a win-win, multi-win, and shared victory for both sports consumers and producers [43, 44].

Thirdly, the dissemination of Internet information has broadened the channels for individual information collection, strengthening social networks [45]. Individuals share sports information and interact with others about sports due to extreme interpersonal communication on social networks [46, 47]. However, this also means that online socialization replaces the social attributes and emotional support of sports, potentially limiting individual sports behavior [48]. Bowby introduced the concept of psychological attachment from the perspective of the relationship between infants and their mothers [49]. Nowadays, the attachment theory refers to the emotional connection between an individual and one or more persons, i.e., someone’s psychological tendency to seek closeness with another for a sense of security [50, 51]. Due to the emergence and development of virtual social platforms, people are influenced by the emotional bonds formed through interactions with others on the Internet, leading to a psychological attachment to the Internet and online friends [52].

**Fig 1:**
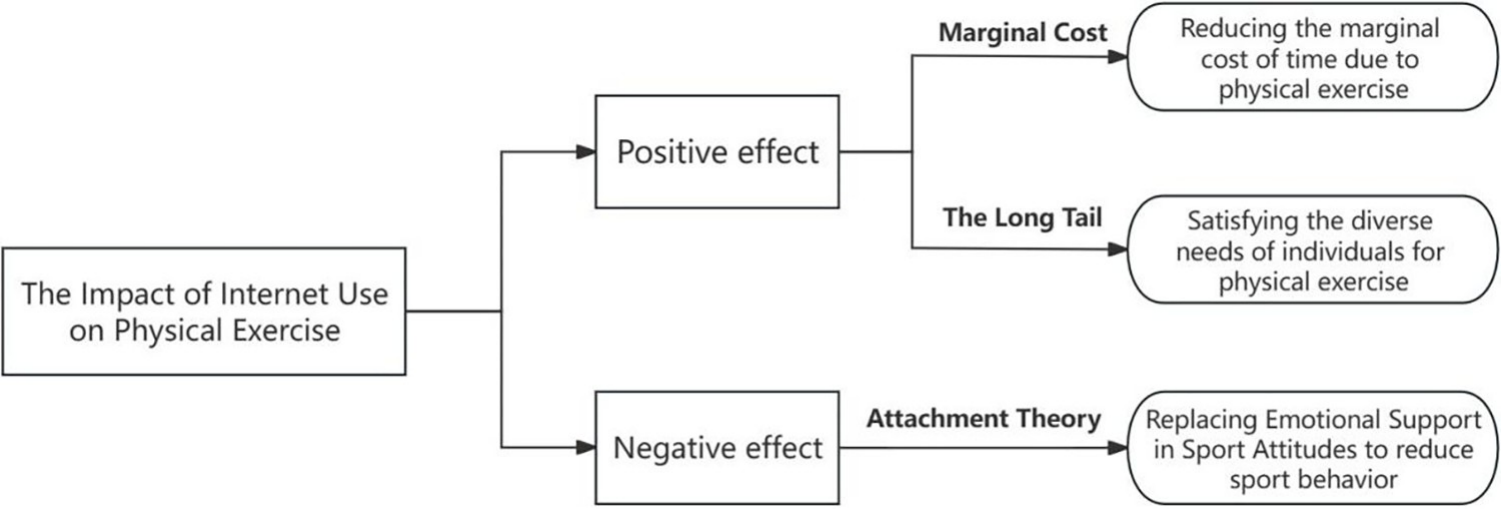
Internet-physical exercise analytical framework.

Based on previous literature, (1) related studies still exhibit some controversy regarding the relationship between Internet use and physical exercise, without fully considering the situation of the middle- and younger-aged population; (2) much consideration is given to the impact of individual factors on physical exercise, with less attention on the influence of other factors related to the Internet; and (3) from a micro perspective, the impact of Internet use on physical exercise among the middle- and younger-aged population has not been fully considered and their associated mechanisms have not been discussed. To address these issues, this study utilized data from the 2018 CFPS to analyze the impact of Internet use on physical exercise among the middle- and younger-aged population from both theoretical and empirical perspectives. Furthermore, this study used empirical testing to examine the internal mechanisms of Internet use in relation to physical exercise based on the three major effects of the Internet (Fig 1). Compared with existing literature, the potential marginal research contributions of this study are as follows:

1. This study focused on the impact of Internet use on physical exercise behaviors among the middle- and younger-aged populations. This group accounts for approximately 70% of the entire Chinese population. Our findings can help with a better understanding of their physical exercise behavior and can assist policymakers in devising effective interventions to positively transform residents’ physical exercise behaviors.
2. We refined the measurement indicators of Internet use and constructed a new theoretical framework. We not only analyzed the impact of usage frequency in the age group on physical exercise but also further explored the relationship between the purpose of use and physical exercise. This multidimensional analysis can provide reference for the government to formulate future policies on Internet use and physical exercise participation.
3. Our research used nationally representative data with a large sample size. As China is a developing country, this study will facilitate comparisons between the survey findings of developing and developed nations.

## 3. Method

This article aims to thoroughly explore how internet use affects physical exercise in the middle-aged and young population, using the 2018 China Family Panel Studies (CFPS) data for individuals aged 15 to 59. It treats the frequency of physical exercise as the dependent variable and internet use as the main independent variable. Given that the frequency of physical exercise is a count variable with varying probabilities of individual events, the paper uses both ordinary least squares and negative binomial regression to build econometric models. To tackle endogeneity, the article employs the individual’s perceived importance of the internet as an instrumental variable in its modeling. Subsequently, the paper delves into how the internet influences physical exercise, drawing on the dual nature of the internet as explained by marginal cost theory, long tail theory, and attachment theory. Finally, the article performs a heterogeneity analysis of physical exercise in the middle- and younger-aged population, focusing on factors like age, household registration, and employment status.

### 3.1 Sources of data and variable configuration

This article uses data from the 2018 China Family Panel Studies by Peking University, covering China’s social, economic, demographic, educational, and health aspects. Data is available at: https://opendata.pku.edu.cn/. In line with this article’s research requirements, referencing the “Explanation on the Age Definition of ’Youth’ and ’Adolescents’” and the “Statistics Law of the People’s Republic of China” [53], we retained samples aged 15 to 59 years. Samples with incomplete or significantly erroneous information were excluded, resulting in 19,588 valid samples, with males making up 50.2%.

This paper focuses on the physical exercise of the middle- and younger-aged population, selecting exercise frequency—the number of weekly exercise sessions—as the proxy variable. Internet use, determined by whether respondents use a mobile phone or computer to access the internet according to the questionnaire, is the primary independent variable, categorized as a binary variable. Control variables encompass individual traits, social characteristics, and occupational traits. Individual characteristics cover gender, age, age squared, education level, household registration type, marital status, health status, and intelligence level. Education level is divided into three ascending categories: up to junior middle school, high school/technical secondary school, and junior college or higher. Household registration types are classified as agricultural and non-agricultural. Marital status is determined by the number of marital stages. Health status is a binary variable, determined by the presence or absence of chronic diseases. Intelligence level is assessed by IQ test scores from the questionnaire. Social network is a continuous variable, measured by scores for "How good are your interpersonal relationships" from the questionnaire, where higher scores indicate better relationships. Social status is divided into five levels, with higher levels indicating greater status. Occupational traits cover employment status. Employment status is valued from 0 to 2, representing unemployed, employed, and out of the labor market, respectively. Table 1 displays the specific variable settings.

**Table 1:** Measurements of variables and descriptive statistics.

	variable	Variable description	Observations	mean(percentage)	standard deviation
dependent variable	Exercise frequency	Count variable, number of workouts per week	19588	2.179	2.994
core independent variable	Internet use	A binary variable, depending on whether you use a mobile phone or computer to access the Internet, yes is set to 1, no is set to 0	19588	0.656	0.475
individual characteristics	gender	Binary categorical variable, male is set to 1, female is set to 0	19588	0.502	0.500
	age	continuous variable	19588	40.269	11.063
	age party	age squared term	19588	1743.966	882.086
	education level	Categorical variables are assigned values in order from low to high education level. Junior high school and below are set to 0, high school / technical secondary school is set to 1, and college and above are set to 2.	19588	0.502	0.770
	Household type	As a two-category variable, agricultural household registration is set to 1 and non-agricultural household registration is set to 2.	19588	1.236	0.425
	marital status	Number of marriages	19588	0.848	0.362
	Health status	Binary categorical variable, whether there is a chronic disease, is set to 1 if it is, and is set to 0 if it is not.	19588	0.119	0.323
	intelligence level	intelligence score	19588	5.174	1.387
social characteristics	social network	Relationship score	19588	7.046	1.889
	Social status	five-level rating	19588	3.000	1.043
Occupational characteristics	employment status	Categorical variables, unemployment is set to 0, being employed is set to 1, and exiting the labor market is set to 2	19588	1.246	0.685

### 3.2 Model construction

**Fig 2:**
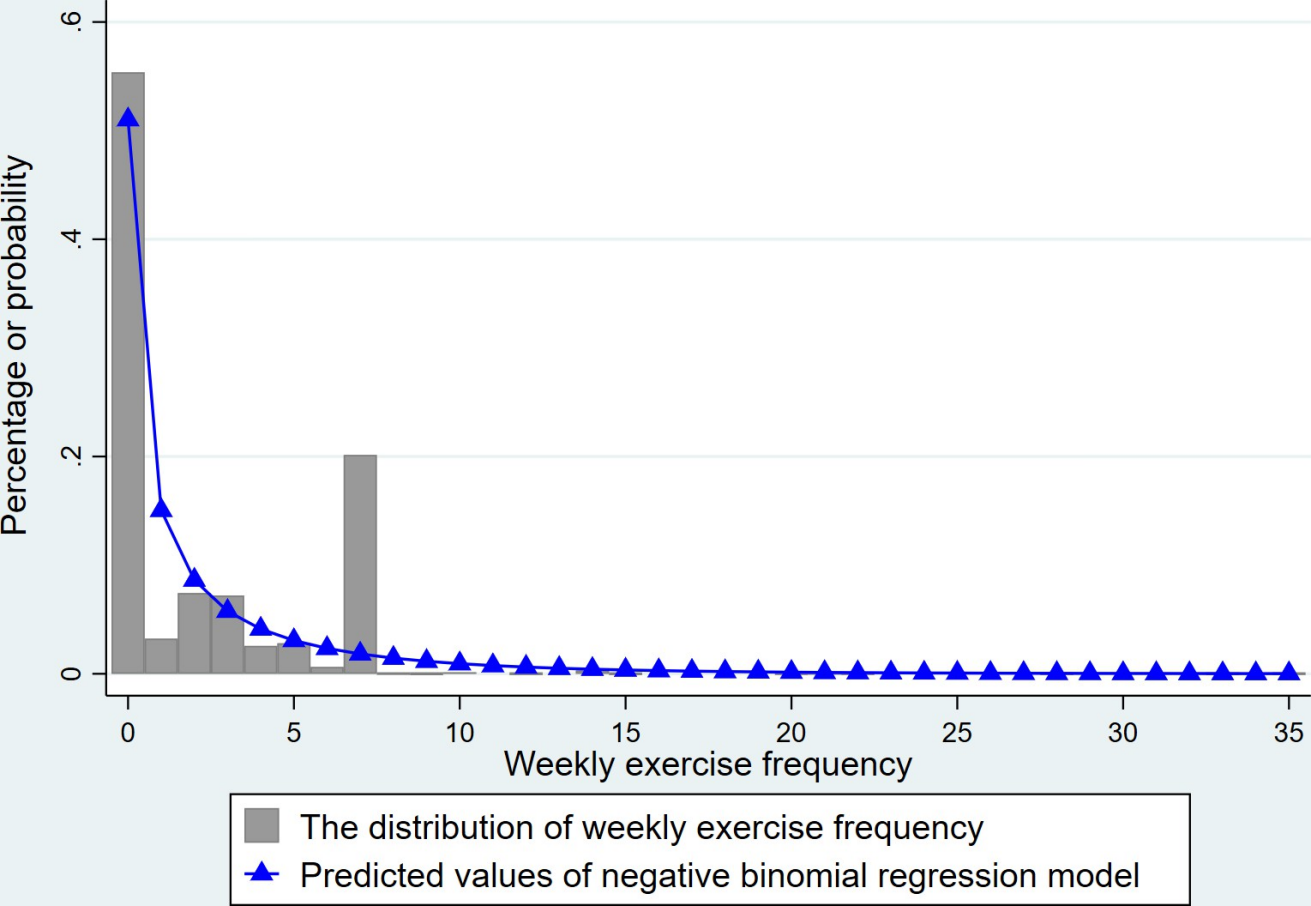
Predicted values of sample distribution and negative binomial regression model.

The dependent variable, the frequency of physical exercise, is a non-negative integer, hence the use of a count model. The Poisson model, the most common count model, assumes the dependent variable follows a Poisson distribution with equal mean and variance—a condition rarely met in reality. However, negative binomial regression effectively addresses "overdispersion" in reality, improving estimate accuracy. The variance of the exercise frequency sample (8.966) is over four times its mean (2.179), and the negative binomial regression model’s predictions align closely with the sample distribution (Fig 2), necessitating negative binomial regression for analysis. Additionally, this paper uses OLS regression for experimental reference and applies the instrumental variable method (IV) to minimize endogeneity issues in the model. The foundational model is established as follows:

$${Exercise}_{i}=\alpha +\beta {Internet}_{i}+\gamma C_{i}+\mu_{i}$$ (1)

In the equation, the dependent variable **Exercise_i_** represents the weekly exercise frequency of the middle- and younger-aged population; the primary independent variable **Internet_i_** represents internet use. Control variables **C_i_** include gender, age, squared age, level of education, type of household registration, marital status, health status, intelligence, social network, social status, employment status, etc. Additionally, **α** represents the constant term, and **μ_i_** represents the error term.

## 4. Results

### 4.1 Descriptive analysis

**Fig 3:**
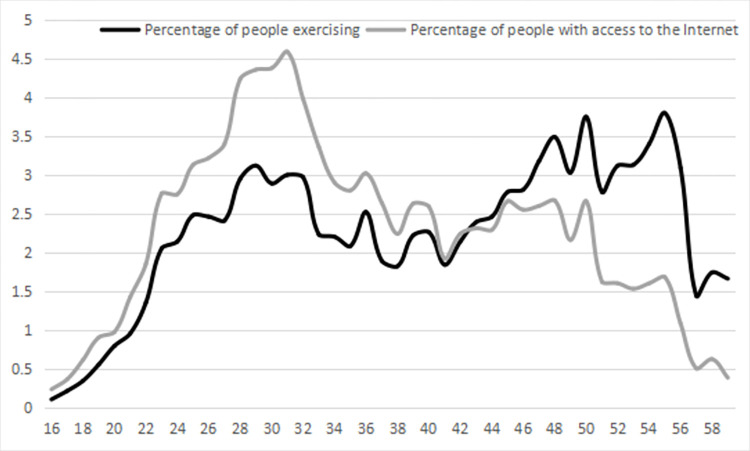
Age-specific distribution of internet use and physical exercise among middle- and younger-aged population (Unit: %).

The analysis of age distribution for internet use reveals that the 28 to 32 years age group has the highest proportion of users. This proportion decreases as age increases. The age distribution of physical exercise shows significant peaks within the 28 to 32 and 48 to 55 age groups (Fig 3). The age distributions for both internet use and physical exercise display similar patterns. Age groups with higher internet usage also show higher participation in physical exercise, suggesting a preliminary positive correlation between internet use and physical exercise.

### 4.2 Regression results analysis

Table 2 displays the regression results for the impact of internet use on physical exercise in the middle- and younger-aged population. The OLS regression reveals a significant positive effect of internet use on exercise frequency. Model 3 focuses exclusively on the impact of internet use on exercise frequency. The results indicate that each unit increase in internet use boosts the exercise frequency to 1.131 times the original amount. Building on Model 3, Model 4 additionally considers individual, social, and occupational characteristics. The findings still indicate a significant positive correlation, with internet use resulting in a 1.303-fold increase in exercise frequency. The Lnalpha value is significantly different at the 1% level, suggesting that the sample is well-suited for negative binomial regression. Future analyses will rely on negative binomial regression results.

**Table 2:** The effect of internet use on the frequency of physical exercise among middle- and younger-aged population.

	OLS regression		negative binomial regression	
	Model 1	Model 2	Model 5	Model 6
	Exercise frequency	Exercise frequency	Exercise frequency	Exercise frequency
Whether to access the Internet	0.264***	0.627***	0.123***	0.264***
	(0.045)	(0.054)	(0.028)	(0.033)
gender		0.137***		0.066**
(Female = 0)		(0.044)		(0.027)
age		-0.038**		-0.005
		(0.017)		(0.011)
age party		0.001***		0.000**
		(0.000)		(0.000)
education level				
(Junior high school and below = 0)				
High school / technical secondary school		0.453***		0.209***
		(0.062)		(0.039)
College degree and above		0.334***		0.167***
		(0.069)		(0.043)
Hukou		0.422***		0.161***
(Rural household registration = 0)		(0.056)		(0.035)
marital status		-0.174**		-0.116***
		(0.068)		(0.044)
disease		0.344***		0.152***
		(0.066)		(0.041)
intelligence level		0.083***		0.038***
		(0.016)		(0.010)
social network		0.076***		0.038***
		(0.012)		(0.007)
Social status		0.124***		0.055***
		(0.021)		(0.013)
employment status				
(unemployed = 0)				
employment		-0.680***		-0.295**
		(0.187)		(0.116)
exit the labor market		-0.385**		-0.180
		(0.195)		(0.121)
Constant term	2.006***	0.021	0.696***	-0.411*
	(0.036)	(0.366)	(0.023)	(0.234)
lnalpha			1.115***	1.060***
			(0.016)	(0.016)
Observations	19588	19588	19588	19588
R ^2^	0.002	0.050	0.000	0.007

Standard deviations are in parentheses.

***p<0.01

**p<0.05

*p<0.1.

### 4.3 Endogeneity discussion

To address potential issues with causality, sample selection, and omitted variables, this paper uses the instrumental variable method to remove model endogeneity. We selected individuals’ perceptions of the internet’s importance as the instrumental variable, reasoning that the greater the perceived importance, the higher the likelihood of its use. This subjective perception, however, is not associated with physical exercise and is therefore uncorrelated with the error term. The instrumental variable method was estimated using both OLS and negative binomial models. Table 3 contains the specific regression results. Model 5 shows Shea’s partial R^2^ at 0.1997 for the first stage of 2SLS regression, and a minimum eigenvalue statistic of 4885.2, above 10, indicating the absence of weak instrument variable issues. Concurrently, the Hausman test yielded a P-value of 1.0000, leading to acceptance of the null hypothesis. Given the traditional Hausman test’s invalidity under heteroskedasticity, we proceeded with a Durbin-Wu-Hausman test. The P-values for both the Chi-square and F-statistics are less than 0.0000, indicating the instrument variable is endogenous. LIML and GMM estimation methods were also used (Model 6 and 7), yielding robust results. Model 8 and 9 result from a two-step method used to address endogeneity issues [54]. The first step involves performing a Logit regression on internet use with the instrumental variable and other controls. The second step entails conducting a negative binomial regression on exercise frequency using the first step’s residuals and control variables. Model 8 demonstrates that the internet’s importance significantly positively affects its use, validating the internet’s importance as an appropriate instrumental variable. Model 9’s regression results reveal significant residuals from Model 8 at the 1% level, with the model passing the joint significance test, suggesting endogeneity in the original model. After adjustments, internet use continues to significantly positively affect exercise frequency among the middle- and younger-aged population, with an increased coefficient. With internet use resulting in a 1.549-fold increase in exercise frequency. This suggests that the internet’s impact on exercise frequency is more significant than initially expected.

**Table 3:** The effect of internet importance on the frequency of physical exercise among youth in physical exercise: IV estimation.

		OLS regression		negative binomial regression	
	Model 5	Model 6	Model 7	Model 8	Model 9
	2SLS	LIML	GMM	Step 1	Step 2
	Exercise frequency	Exercise frequency	Exercise frequency	Whether to use the Internet	Exercise frequency
importance of internet	0.460***	0.460***	0.460***	0.731***	
	(0.035)	(0.035)	(0.036)	(0.015)	
Whether to use the Internet					0.466***
					(0.043)
Model 8 residual value					-0.097***
					(0.016)
control variables	Controlled	Controlled	Controlled	Controlled	Controlled
Constant term	-0.688*	-0.688*	-0.688*	-1.698***	-0.498***
	(0.379)	(0.379)	(0.366)	(0.404)	(0.180)
lnalpha					1.057***
					(0.016)
Observations	19588	19588	19588	19588	19588
R ^2^	0.032	0.032	0.032	0.414	0.007

Standard deviations are in parentheses. Control variables include 11 variables including individual characteristics, social characteristics, and occupational characteristics.

***p<0.01

**p<0.05

*p<0.1

### 4.4 Analysis of the mechanism of internet use on physical exercise among middle- and younger-aged populations

After clarifying the positive impact of Internet use on physical exercise among the middle- and younger-aged populations, this study further explored the mechanisms through which Internet use affects physical exercise. The previous sections discussed the dual nature of the effect of the internet on physical exercise based on the marginal cost, long-tail, and attachment theories. Therefore, we chose individual online learning and entertainment as the positive effects of the Internet, with online socialization as the negative effect of the internet, to examine the intrinsic mechanisms of the internet’s impact on physical exercise.

This study targets middle- and younger-aged Internet users as participants. Based on questions from the 2018 CFPS questionnaire regarding the frequency of using the Internet, three indicators, including online learning, entertainment, and online socialization, served as proxies to assess the Internet’s positive and negative effects and a regression model was constructed, with specific results presented in Table 4.

**Table 4:** Analysis of the role mechanism of online learning, online entertainment, and online socialization.

	OLS regression		negative binomial regression	
	Model 10	Model 11	Model 12	Model 13
	Exercise frequency	Exercise frequency	Exercise frequency	Exercise frequency
online learning	0.131***	0.135***	0.057***	0.063***
	(0.011)	(0.012)	(0.006)	(0.007)
online entertainment	-0.007	0.044***	-0.001	0.018**
	(0.015)	(0.014)	(0.009)	(0.009)
online socialization	-0.090***	-0.038**	-0.040***	-0.023**
	(0.016)	(0.016)	(0.009)	(0.009)
control variables	Uncontrolled	Controlled	Uncontrolled	Controlled
Constant term	2.453***	0.952**	0.886***	-0.185
	(0.091)	(0.441)	(0.052)	(0.263)
lnalpha			0.891***	0.818***
			(0.020)	(0.020)
Observations	12843	12843	12843	12843
R ^2^	0.012	0.072	0.002	0.010

Standard deviations are in parentheses. The control variables in Models 11 and 13 include 11 variables including individual characteristics, social characteristics, and occupational characteristics.

***p < .0.01

**p<0.05

*p<0.1.

Initially, online learning emerged as the principal mediator enhancing physical exercise among middle- and younger-aged individuals, confirmed using OLS and negative binomial regression analyses. The results from Model 13 indicated that for every unit increase in online learning, the frequency of physical exercise increased 0.063. Subsequently, upon adjusting for individual attributes, social factors, and professional characteristics, online entertainment positively affected exercise frequency among middle- and younger-aged individuals, the frequency of physical exercise increased 0.018. Its significance and coefficient are lower than those of online learning. All models consistently revealed a significant adverse effect of online socialization on individual physical exercise, for every unit increase in online socialization, the frequency of physical exercise reduced 0.023.

Although online socialization’s negative effects partially counteract the positive impacts of online learning and entertainment, the Internet’s positive influences on physical exercise among the middle- and younger-aged population significantly outweigh its negatives.

### 4.5 Analysis of the heterogeneity in physical exercise among middle- and younger-aged populations

This study conducted a heterogeneity analysis of physical exercise among middle- and younger-aged populations based on age, household registration type, and employment status, with specific results shown in Table 5.

**Table 5:** Analysis of the heterogeneity of internet use on physical exercise among middle- and younger-aged populations: A negative binomial regression.

	Model 14	Model 15	Model 16	Model 17	Model 18	Model 19	Model 20
	35 years old and	Over 35 years old	Agricultural household registration	Non-agricultural	unemployment	Working	exit the labor market
	the following			Hukou			
	Exercise frequency	Exercise frequency	Exercise frequency	Exercise frequency	Exercise frequency	Exercise frequency	Exercise frequency
Whether to use the Internet	0.045	0.315***	0.260***	0.250***	0.322	0.280***	0.196**
	(0.075)	(0.039)	(0.041)	(0.061)	(0.254)	(0.036)	(0.093)
control variables	Controlled	Controlled	Controlled	Controlled	Controlled	Controlled	Controlled
Constant term	-1.237	-1.638	-0.276	-0.169	0.369	-0.521**	-1.591***
	(0.760)	(1.105)	(0.294)	(0.391)	(1.241)	(0.233)	(0.551)
lnalpha	1.030***	1.069***	1.283***	0.365***	0.422***	1.054***	1.138***
	(0.027)	(0.021)	(0.019)	(0.034)	(0.147)	(0.018)	(0.045)
Observations	7428	11687	14967	4621	249	16807	2532
R ^2^	0.004	0.008	0.005	0.011	0.019	0.006	0.012

Standard deviations are in parentheses. The control variables in Models 14 and 15 include 11 variables including individual characteristics, social characteristics, and occupational characteristics. The control variables in Models 16 and 17 include 10 variables including individual characteristics, social characteristics, and occupational characteristics. The control variables in Models 18–20 include 10 variables including individual characteristics and social characteristics.

***p<0.01

**p<0.05

*p<0.1.

Using 35 years as the threshold, this study found that Internet usage does not significantly affect physical exercise among those aged 35 years and below but has a significant impact on those above 35 years. Model 15 shows that among the population over 35 years, with internet use resulting in the frequency of physical exercise increased to 1.37 times. Model 16 and 17 show that Internet use had a positive effect on physical exercise among middle- and younger-aged populations with different household registration types, for the agricultural household registration group, the frequency of physical exercise will increase by 0.260, whereas for the non-agricultural household registration group, it will increase by 0.250. Internet use had a positive effect on physical exercise with who are employed or have exited the labor market, with an observed increase by 0.280 and 0.196, respectively. There is no significant impact on the unemployed group.

## 5. Discussions

Initially, our findings revealed that middle- and younger-aged individuals, as key participants in Internet usage and physical exercise, can benefit positively from the Internet’s impact on physical activity. Concerning the link between Internet usage and physical exercise, prior research has yielded two distinct conclusions. Firstly, Internet usage can enhance individuals’ engagement in physical activities. For instance, the Chinese General Social Survey data from China demonstrate that residents who regularly used the Internet partook in considerably more physical activity [55]. Comparable outcomes have been verified in Europe [56]. Secondly, Internet usage has a suppressive effect on individuals’ participation in physical exercises. For instance, the Internet can result in sedentary behaviors among individuals, subsequently diminishing their participation in physical activities [57]. Additional research has also indicated that lower physical activity levels are linked to Internet usage [58]. Our study’s findings endorse the first perspective.

Secondly, regarding the mediating mechanisms, online learning and entertainment promote physical exercise among young and middle-aged adults, while online socialization inhibits participation. Our study diverges from previous views [49] and according to the Dual Process Theory, posits that online learning involves explicit, conscious processes, while online entertainment and online socialization involve implicit (automatic), unconscious processes. Online learning is purposeful, leading young and middle-aged adults to deliberately and consciously increase their physical exercise participation. Online entertainment intuitively and emotionally promotes physical exercise in young and middle-aged adults because it can trigger dopamine release and enhance subjective well-being. Online socialization, due to its implicit, unconscious, emotional attachments and its nature of networked individualism, leads young and middle-aged adults to spend more time on the Internet. It replaces the emotional dimension of attitudes toward sports and the social attributes of sports and makes people more likely to alleviate social anxiety and loneliness directly through online social interactions rather than fulfilling their psychological needs through social interactions during physical exercise [59].

Lastly, we also found that Internet use has a more pronounced impact on the younger- and middle-aged population over 35 years of age. According to the Health Belief Model, people aged over 35 years have deeper self-awareness and are more adept at using the Internet to obtain health information and improve their health level [60, 61]. This indicates that the Internet can reduce the health disparities among residents of different ages. Additionally, Internet use had a significant positive effect on physical exercise among the younger- and middle-aged population who are employed or have exited the labor market, with increases by 0.280 and 0.196, respectively. However, no significant effect was observed among the unemployed younger- and middle-aged population. We believe this is because the fitness participation of the unemployed group faces challenges such as a lack of positive mindset and favorable economic conditions, absence of scientific guidance, and lack of facilities. Therefore, the impact of the Internet on their physical exercise participation is very limited.

Based on our findings, we propose the following policy recommendations to enhance the role of the Internet in encouraging the participation of younger- and middle-aged groups in physical exercise. Firstly, regarding emotional attachment in online socialization, we recommend that the government utilizes new media platforms such as TikTok and YouTube to lead physical exercise initiatives [62]. Regarding social capital, these platforms reduce the digital divide among different young and middle-aged individuals and transform the viewing model of physical exercise guidance into interactive forms such as bullet comments, check-ins, and connections [63]. Young and middle-aged individuals can discover a sense of self-presence in physical exercise on social networking platforms. Secondly, regarding the physical exercise age boundary of 35 years observed among the younger- and middle-aged population, we suggest that the government disseminates more scientific fitness knowledge through the Internet to facilitate active fitness awareness among the youth under 35 years old. The population below 35 years old relatively lack self-discipline [64]; thus, in formulating related policies, it is crucial to consider the distribution of Internet and sports resources among the youth under 35 years to further promote a balance between Internet services and public sports facilities for different age groups [65]. Lastly, as the unemployed younger- and middle-aged population are unable to significantly benefit from Internet use in terms of physical exercise participation, we recommend that the government focuses on the health behaviors of the unemployed. Unemployed individuals, having lost income and the opportunity to work, do not only lose material resources but also suffer in terms of intangible gains [66]. This includes losing a structured daily routine, severing ties with colleagues in previous workplaces, etc. The low subjective well-being and increased feelings of loneliness among such individuals necessitate focused government attention, including the provision of services such as sports medicine consultations provided by the government.

## 6. Limitations

This study has certain limitations. Firstly, this study assessed the impact of Internet use on physical exercise among the middle- and younger-aged population using a cross-sectional design. The study lacks longitudinal analysis, particularly in terms of the physical activity status of Chinese residents after experiencing the COVID-19 pandemic isolation policies. In the future, we will collect empirical data to further explore the impact of Internet use on physical exercise among middle- and young-aged people in the post-COVID-19 era. Secondly, compared with previous studies, although we further limit the study population to the middle- and younger-aged population and identify the inhibitory role of online socialization, differentiation in the specific manifestations of different mediating effects across various age groups is lacking. In the future, we plan to investigate research indicators related to the population under 35 years old for further exploration and explanation.

## 7. Conclusions

Prior research has primarily focused on how Internet use affects the physical and psychological health of the elderly and youth, with little systematic examination of the relationship and mechanisms between Internet use and sports participation in the middle- and younger-aged population. Based on the CFPS2018 data, this study empirically analyzed the relationship between internet use and physical exercise among middle- and younger-aged people in the context of a digital society, summarizing the positive and negative effects of the Internet on physical exercise using the marginal cost, the long tail, and attachment theories. We found that (1) Internet use significantly increases the frequency of physical exercise among middle- and younger-aged populations, with each unit increase in individual internet use increasing the frequency of physical exercise to 1.549 times its original value; (2) Currently, the positive effects of the Internet outweigh its negative effects, promoting individual physical exercise effectively. The positive effects of the Internet were primarily realized through online learning and entertainment, which increased the frequency of physical exercise among the middle- and younger-aged population by 0.063 and 0.018, respectively. The adverse effects of the Internet manifested through online social interactions, reducing the frequency by 0.023; and (3) The influence of Internet usage is notably greater among individuals aged over 35 years, with internet use resulting in the frequency of physical exercise increased to 1.37 times. Moreover, Internet use significantly positively influenced physical exercise among the middle- and younger-aged populations who were employed or had exited the labor market, demonstrates the robustness of the obtained results.
