# Correction: Relationship and mechanisms between internet use and physical exercise among middle- and younger-aged groups

**DOI:** 10.1371/journal.pone.0307926

**Published:** 2024-07-24

**Authors:** The PLOS ONE Staff

The published funding statement is incorrect. The publisher apologizes for this error.

The correct funding statement is: This work was supported by the National Social Science Fund of China, "A Study on the Income Risk of Rural Residents’ Health under the Background of Healthy China" (Grant No. 20BRK005). The funders had no role in study design, data collection and analysis, decision to publish, or preparation of the manuscript.
